# Hemodialysis Adequacy and Its Impact on Long-Term Patient Survival in Demographically, Socially, and Culturally Homogeneous Patients

**DOI:** 10.1155/2020/9857123

**Published:** 2020-08-19

**Authors:** Reza Hekmat

**Affiliations:** Mashhad University of Medical Sciences, Ghaem Hospital, Department of Nephrology, Mashhad, Iran

## Abstract

**Background:**

Impact of hemodialysis adequacy on patient survival is extensively studied. The current study compares the survival of chronic hemodialyzed, undocumented, uninsured, Afghan immigrant patients with that of a group of insured Iranian patients matched for underlying disease, age, weight, level of education, marital status, income, and number of comorbid conditions.

**Methods:**

Eighty chronic hemodialysis patients (mean age 42.8 ± 10.5 years) entered this historical cohort study in Mashhad, Iran, between January 2012 and January 2015. Half of the patients were undocumented, uninsured, Afghan immigrants (Group A) matched with forty insured Iranian patients (Group B). To compare the survival rate of the two patient groups, Kaplan–Meir survival analysis test was used.

**Results:**

Group A patients were underdialyzed with a weekly *Kt*/*V* which was significantly less in comparison with that of Group B (1.63 ± 0.63 versus 2.54 ± 0.12, *p* value = 0.01). While Group A's number of hemodialysis sessions per week was fewer than that of Group B (1.45 ± 0.56 versus 2.8 ± 0.41, *p* value = 0.04), the mean of *Kt*/*V* in each hemodialysis session was higher in them, in comparison with Group B (1.43 ± 0.25 versus 1.3 ± 0.07, *p* value = 0.045). In Group B and Group A patients, one-year survival was 70% versus 50%, two-year survival was 55% versus 30%, and three-year survival was 40% versus 20%, respectively (*p* values = 0.04, 0.02 and 0.04, respectively). In Cox regression analysis, hemodialysis adequacy and uninsurance were factors impacting patients' survival (OR = 1.193 and 0.333, respectively).

**Conclusions:**

Undocumented, uninsured, inadequately hemodialyzed, Afghan patients had a significantly lower one-, two-, and three-year survival as opposed to their Iranian counterparts, probably due to lack of insurance.

## 1. Introduction

There is an undisputable relation between hemodialysis adequacy and patient survival. The question of the optimal level of hemodialysis for the highest patient survival remains unanswered [1, 2]. Health care disparity can be defined as the difference in the accessibility of health care facilities and services among a racially and/or ethnic and/or geographically and/or politically defined group of people [3–5]. Although many studies have addressed racial/ethnic disparities in health in the United States, the issue of uninsurance, as a health disparity, has not been addressed in immigrant undocumented Afghan hemodialyzed patients. Against a matched group of Iranian hemodialysis patients with adequate funding for their treatment, the current study compared the adequacy of the hemodialysis and survival of undocumented, uninsured, Afghan chronic hemodialyzed patients who were illegal immigrants in Iran. Deemed as illegal residents, these patients were not officially recognized by the United Nations High Commission for Refugees. As a result of their nonstatus, they suffered from financial problems and a lack of medical insurance coverage during the time period of January 2012 to January 2015. Some did receive financial aid for part of their hemodialysis expenses from charities. Several studies in the United States have reported a correlation between the absence of health insurance and residency in a high-poverty area with marked delays in renal replacement therapy initiation in ethnic minorities [6, 7]. In addition, the level of public investment in health has been found to seriously impact every aspect of kidney disease so that even the estimation of end-stage renal disease incidence varies greatly among the different regions of the world [8]. A racially and/or ethnic and/or geographically and/or politically defined group of persons may experience health care disparity when there is a palpable disadvantage for such a group when attempting to access health care [3–5]. Other than various studies on racial/ethnic-based disparities in American health care, there have been no papers addressing this issue as it exists in the contemporary sociopolitical environment of the Middle East. Several U.S.-based works have discussed the relationship between a lack of medical insurance and inhabiting a high-poverty area, which manifests itself in significant delays in renal replacement therapy initiation among ethnic minority groups [3, 6, 7]. In this study, we tried to evaluate the effect of presumably inadequate hemodialysis on patient survival, and then, we addressed the possible impact of the demographic, social, cultural, and economic conditions of patients, especially insurance coverage and the related health disparity on the inadequacy of hemodialysis and subsequently patient survival.

## 2. Methods

From January 2012 to January 2015, 80 chronic hemodialysis patients (Table 1) participated in this historical cohort study conducted in Mashhad, Iran. So as to comply with the selected time frame, from January 2012, Iranian patients were retrospectively selected from a larger cohort of about 500 patients in order to do one-to-one matching with undocumented, uninsured, Afghan patients. A longitudinal study is a design for research which involves repeated observation of the same variables (e.g., measurements and people) over a long or short duration of time [8]. Half of the study patients were undocumented, uninsured, Afghan immigrants (Group A) and the other half were Iranians (Group B). In the time period between January 2012 and January 2015, regular and adequate hemodialysis was not affordable for Group A patients as they were uninsured. We hypothesized that this health insurance dichotomy may have culminated in chronic inadequate hemodialysis in these patients. On the first visit, the following patients were excluded from the present study: patients with recognized active infection and/or arteriovenous or catheter malfunction. Also excluded were those patients suffering from severe heart failure and/or depression. The two study groups were matched for underlying disease, age, weight, level of education, marital status, income, number of comorbid conditions, and the time duration of hemodialysis from its start (Table 1). More than 80% of the patients in both groups were officially illiterate, and the monthly income at a common currency exchange rate, for both groups, was less than USD $58, which is the internationally recognized absolute poverty line. All patients were from the same municipal district of the city. Also, this fact that the majority of patients in both groups were married may have increased the economic burden on them and their families. As suggested by KDOQI clinical practice guideline for hemodialysis adequacy [9, 10], for thrice-weekly hemodialysis schedule, the target single pool *Kt*/*V* (*K*: dialyzer clearance of urea, *t*: dialysis time, and *V*: volume of distribution of urea) of 1.4 per session with a minimum delivered sp*Kt*/*V* of 1.2 was defined as the standard adequate hemodialysis. The blood flow rate, dialyze mass transfer coefficient (KoA), dialysate flow, and needle size were also matched between the two patient groups, and adequate anticoagulation was provided for all patients [11]. The Kaplan–Meir survival analysis was utilized for comparing the survival rate of the two groups. Chi-square statistics and paired student *t*-test were used for evaluating the categorical and continuous parameters, respectively, between two groups. The Statistical Package for the Social Sciences software (SPSS version 20.0, SPSS Inc., Chicago, IL, USA) was used for data analyses. A *p* value of <0.05 was considered significant. The study was approved as a clinical study by the Ethical Committee and Research Council of Mashhad University of Medical Sciences (code numbers: 6856226 and 87359, respectively).

## 3. Results

**Figure 1:**
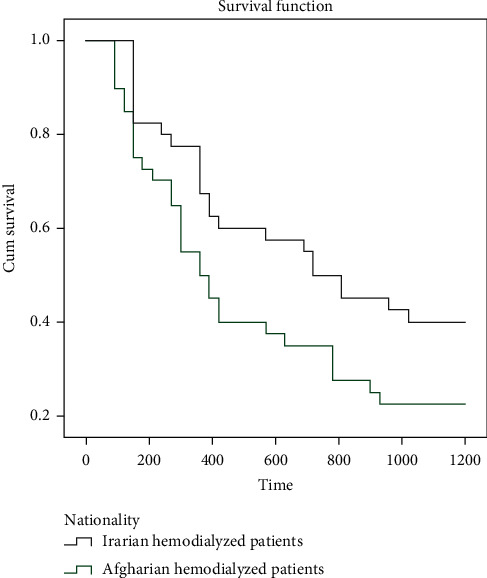
The Kaplan–Meier survival plot shows more longevity in chronically hemodialyzed Iranian patients compared with their less adequately hemodialyzed Afghan counterparts.

Some basic demographic and social characteristics of two groups, including age and gender, are depicted in Table 1. Group A patients were underdialyzed, with higher predialysis BUN values and a weekly *Kt*/*V* significantly less than that of group B (Table 2). While the number of hemodialysis sessions per week was fewer in group A than that of in group B (Table 2), the mean of single pool *Kt*/*V* in each hemodialysis was higher in group A compared to that in group B (Table 2). The Kaplan–Meir survival analysis reported lower three years longevity among the inadequately hemodialyzed, undocumented, uninsured, Afghan patients, in comparison with the more adequately hemodialyzed Iranian patients (logrank test value = 0.04) (Table 3, Figure 1). Furthermore, Group A patients were significantly more anemic but not more hyperkalemic or hyperphophatemic than Group B (Table 2). As predicted somehow by design and matching between two groups, throughout the study, we found no statistically significant difference between two groups regarding other demographic, social, economic, and cultural factors; unemployment rate, access to nephrology care before initiating renal replacement, reported satisfaction with health care providers, religion, ownership of residency location, and nutrition status as assessed by nutritional indicators (body mass index, and serum albumin) (Table 4). Table 3 provides the differential causes of death and survival rate for both groups, as depicted, and Group A patients had significantly less 1, 2, and 3 year survival compared with group B patients. In Cox proportional hazards regression analysis, the quantity of hemodialysis as assessed by weekly *Kt*/*V* and insurance status emerged as the only factors impacting survival in two groups (Table 5).

## 4. Discussion

The mean of *Kt*/*V* in each hemodialysis session has been significantly more in Afghan patients compared with their Iranian counterparts; thus, technical failures such as arteriovenous fistula malfunction, can be excluded as a cause of less adequate hemodialysis in this group of patients [12, 13]. This may be due to higher initial BUN pre-HD in group A, but also probably can indicate the impact of Group A's prolongation of hemodialysis sessions, in hope of compensating for their fewer weekly hemodialysis sessions. The survival rate in both groups was lower than the median national rate of more than a 50% four-year survival (according to personal communications with the Iranian Society of Nephrology). In spite of a reported strong correlation between health disparity and residential conditions (e.g., juvenile delinquency rate, unliterary, unemployment rate, recreation facilities, and cultural norms) [14], all of the selected study cases resided in neighborhoods sharing the same conditions. As discussed in some studies, an association exists between decrement in *Kt*/*V* and increased hospitalizations, lengthier hospital days, and higher inpatient insurance costs [15]. Lack of access to high quality nephrology care before initiating renal replacement therapy is linked to the hemodialysis outcome [16–18]. Although poverty level [19], race [3, 16, 17], religion [20], gender [21, 22], health care provider's beliefs, or behavior [23, 24] has been proposed as factors contributing to health disparity, however, in these respects, there was no difference between the two study groups (Tables 2 and 4). During the study's time period, only one patient from each group had underwent a kidney transplant, but returned immediately to hemodialysis due to acute allograft rejection, thus rejecting the idea of a selection bias against Afghan patients. This fact rules out racial or ethnic survival discrimination due to kidney transplantation disadvantage [25–27]. Though some studies have reported a better outcome with the use of an arteriovenous fistula during the first outpatient hemodialysis session [22, 28], only 15% and 18% of Group A and B patients, respectively, utilized an AV fistula during the initial hemodialysis session, thus indicating poor predialysis nephrologic care for both groups (Table 4). All patients had a body weight of less than 72 kilograms, ruling out the possibility of a urea distribution volume (*V*) greater than 40 liters as a possible cause of inadequate hemodialysis [29]. In the study's participating hemodialysis centers, more than 55% of the patients were not Iranians, but Afghans or Arabs. Such an ethnically mixed patient population may itself adversely impact the survival of all patients [30]. Effect of infectious complications' minimization or cardiovascular event prevention, on patient survival is suggested by others [31]; in this study, a number of infectious complications or cardiovascular events culminating to death were numerically, but not statistically, more in undocumented, uninsured, Afghan patients, and this may be due to the small sample size, that is one of our study's limitations. Some researchers have recommended gradual, incremental, or twice weekly hemodialysis for overcoming resource constraints with no effect on the quality of care [32–34]; we were not able to test these options in this study. However, the final word and important limitation of study may be that the present study is not able to establish a direct correlation between hemodialysis inadequacy and survival because lack of insurance and funding, resulting in health disparity, may have deeply impacted other aspects of these patients' health care, thus resulting in lower survival.

## 5. Conclusions

Undocumented, uninsured, Afghan patients with inadequate hemodialysis had a significantly lower survival rate during the three years of the study, compared with the Iranian matched group who received more adequate hemodialysis. Skipping hemodialysis sessions because of inadequate funding and lack of insurance coverage is the most probable cause of this health disparity. Regarding the young mean age of the selected patients, this is a very poor outcome and reflects significant health disparity.

## Figures and Tables

**Table 1:** Some basic demographic and social and economic characteristics of two groups that were matched from the beginning of the study.

Group	Group A	Group B	*p* value

Number of patients	40	40	=1
Age (years)	37.87 ± 9.98	41, 5 ± 10.80	=0.10
Weight (kilograms)	44.50	50.40	=0.79
Male gender (percent)	22/(55%)	22/(55%)	=1
Number of patients with less than 58$ monthly income (percent)	40 (100%)	40 (%100)	=1
Number of illiterate patients	34 (85%)	36 (90%)	=0.50
Current marital status (married/unmarried)	32/8	30/10	=0.8
Time duration from the beginning of hemodialysis (months)	44.05 ± 5.80	50.80 ± 4.80	=0.70
Number of uninsured patients	40	0	<0.01
Diabetes mellitus as the cause of end stage renal disease	4 (10%)	5 (12.5%)	=0.28

**Table 2:** Some hemodialysis characteristics and laboratory parameters in two groups.

Group	Group A	Group B	*p* value

Mean of blood hemoglobin (gram/dl)	7.1 ± 1.4 versus	10.5 ± 1.1 gram/dl	<0.01
Mean of serum potassium (mEq/L)^*∗*^	4.8 ± 1.1	5.1 ± 1.2	=0.68
Mean of serum phosphate (mg/dl)^*∗∗*^	5.86 ± 0.90	6.36 ± 0.81	=0.25
Mean of serum creatinine (mg/dl)^*∗∗*^	5.40 ± 058	5.02 ± 0.49	=0.24
Mean of serum BUN (mg/dl)^*∗∗*^	75.53 ± 35.71	68.15 ± 36.01	<0.01
Mean of weekly *Kt*/*V*	1.63 ± 0.63	2.54 ± 0.12	<0.01
Mean of the numbers of hemodialysis sessions per week	1.45 ± 0.56	2.8 ± 0.41	<0.04
Mean single pool *Kt*/*V* in each hemodialysis	1.43 ± 0.25	1.3 ± 0.07	<0.04
Mean of hemodialysis session length (hours)	4.46 ± 0.42	3.4 ± 0.32	<0.01

^*∗*^Milliequivalents/liter. ^*∗∗*^Milligram/deciliter.

**Table 3:** Cause of death and survival rate in two groups from 2012 to 2015.

Presumed cause of death	Group A	Group B	Total	*p* value between 2 groups

Catheter sepsis or hemorrhage (percentile)	5 (12.5%)	2 (5%)	7 (8.75%)	=0.20
Cardiac arrhythmia (percentile)	2 (5%)	2 (5%)	4 (5%)	=0.69
Acute myocardial infarction (percentile)	4 (10%)	6 (15%)	10 (12.5%)	=0.28
Acute cerebrovascular accident	6 (15%)	5 (12.5%)	11 (13.75%)	=0.50
Severe pneumonia	6 (15%)	4 (10%)	10 (12.5%)	=0.30
Acute gastrointestinal bleeding	3 (7.5%)	0	3 (3.75%)	=0.66
Acute abdomen	3 (7.5%)	0	3 (3.75%)	=0.12
Gangrene of lower extremity	1 (2.5%)	0	1 (1.25%)	=0.50
Disseminated tuberculosis	2 (5%)	0	2 (2.5%)	=0.24
Leukemia	0	1 (2.5%)	1 (1.25%)	=0.50
Gastrointestinal malignancy	0	4 (10%)	4 (5%)	=0.05
Cumulative mortality	32 (80%)	24 (60%)	56 (70%)	=0.02
1-year survival rate	16 (40%)	28 (70%)	44 (55%)	=0.04
2-year survival rate	12 (30%)	22 (55%)	34 (42%)	=0.02
3-year survival rate	8 (20%)	16 (40%)	24 (%30)	Logrank = 0.04

**Table 4:** Some demographic, cultural, medical, social, and economic characteristics of two groups that were discerned during the study.

Group	Group A	Group B	*p* value

Religion (Shiite Moslems)	40 (100%)	40 (100%)	=1
Satisfactions with heath care provider	38 (95%)	36 (90%)	=0.33
Unemployment rate	30 (75%)	32 (80%)	=0.39
Lack of access to high quality nephrology care before initiating renal replacement therapy	28 (70%)	30 (75%)	=0.40
Ownership of residency location	0 (0%)	1 (0.25%)	=0.55
Body mass index (BMI) (kg/m^2^)	22.5 ± 3.19	21.04 ± 2.94	=0.64
Serum albumin (g/dl)^*∗*^	3.41	3.35	=0.29

^*∗*^Gram/deciliter.

**Table 5:** Effect of the dependent variables; hemodialysis adequacy; and insurance status on the dependent variable death-censured graft survival as obtained by Cox's proportional hazards model.

Parameter	Odds ratio	95% confidence interval	Significant	Exp (*B*)

Hemodialysis adequacy	1.193	1.011–1.59	*p* < 0.01	3.298
Uninsurance status	0.333	0.21–0.83	*p* < 0.01	1.395

## Data Availability

The clinical study data used to support the findings of this study are available from the corresponding author upon request.
